# A Case of Parasitic Leiomyoma in the Sigmoid Mesentery Following Total Laparoscopic Hysterectomy

**DOI:** 10.7759/cureus.73940

**Published:** 2024-11-18

**Authors:** Taro Miyake, Yuichiro Tateno, Hiroko Matsuura, Tadashi Aoyama, Kazuya Kudo

**Affiliations:** 1 Obstetrics and Gynecology, Tama-Hokubu Medical Center, Higashimurayama, JPN

**Keywords:** hysterectomy, laparoscopy, leiomyoma, morcellation, parasitic leiomyoma

## Abstract

Parasitic leiomyoma (PL) develops when fragments of a morcellated uterine leiomyoma, during procedures such as laparoscopic myomectomy (LM) or total laparoscopic hysterectomy (TLH), adhere to other tissues. We recently encountered a case where PL developed in the mesentery of the sigmoid colon following TLH. A 51-year-old woman had previously undergone TLH with in-abdominal morcellation. Six years post surgery, she presented with a new mass in her lower abdomen. MRI showed a large tumor (21×12×15 cm) in the pelvic cavity, initially suspected to be a leiomyoma or potentially a malignant tumor, such as a sarcoma or gastrointestinal stromal tumor (GIST), due to its size and unusual location. During the tumor resection, the tumor was found adhering to the sigmoid colon mesentery. Histopathological examination confirmed it was a benign leiomyoma, and the patient’s recovery was uneventful. For large PL, differential diagnosis can be challenging. Demonstrating a solid tumor of unknown cause after gynecologic surgery, especially after myomectomy or myoma morcellation, a parasitic myoma must be included in the differential diagnosis. It is crucial to conduct thorough preoperative evaluations and ensure informed consent by discussing the potential for malignancy and the corresponding treatment options.

## Introduction

Leiomyomas are benign tumors commonly found in the uterus but can rarely occur in extrauterine locations. These ectopic or "parasitic" leiomyomas (PLs) have been reported in various sites, including sigmoid mesentery [1]. PLs are often associated with previous gynecologic procedures, such as laparoscopic myomectomy (LM) or total laparoscopic hysterectomy (TLH). To retrieve leiomyomas through the trocar, the specimen must be morcellated within the abdominal cavity. Fragments of leiomyomas generated by this procedure may remain in the abdominal cavity and develop into PL [2-4]. PL is a rare complication of laparoscopic surgeries involving morcellation, with prevalence estimates ranging from 0.9% to 1.2% [5]. A systematic review identified 274 cases of PL, with 39% of patients having a history of power morcellation [6].

Since the first report of PL following LM in 1997 [7], the risks associated with morcellator use have been closely examined. In-bag morcellation (IBM) seems to eliminate the risk of dispersal of leiomyoma fragments completely; however, Solima et al. reported that minor bag ruptures occur in approximately one-third of all cases [8]. According to an FDA report, uterine sarcoma is mistakenly operated on as a leiomyoma at a rate of one in 225 to one in 580 cases [9]. Therefore, conducting thorough preoperative evaluations and carefully considering the suitability of laparoscopic surgery are crucial, and sharing this information with patients is essential for informed decision-making.

PL can be challenging to diagnose and may mimic other conditions such as ovarian carcinoma or gastrointestinal stromal tumors (GISTs). Management typically involves surgical excision. Accurate diagnosis is crucial, as malignant variants like leiomyosarcomas can occur in similar locations and require different treatment approaches [10].

We present a case of PL that developed in the sigmoid mesentery following TLH. We report this case along with a literature review.

## Case presentation

**Figure 1:**
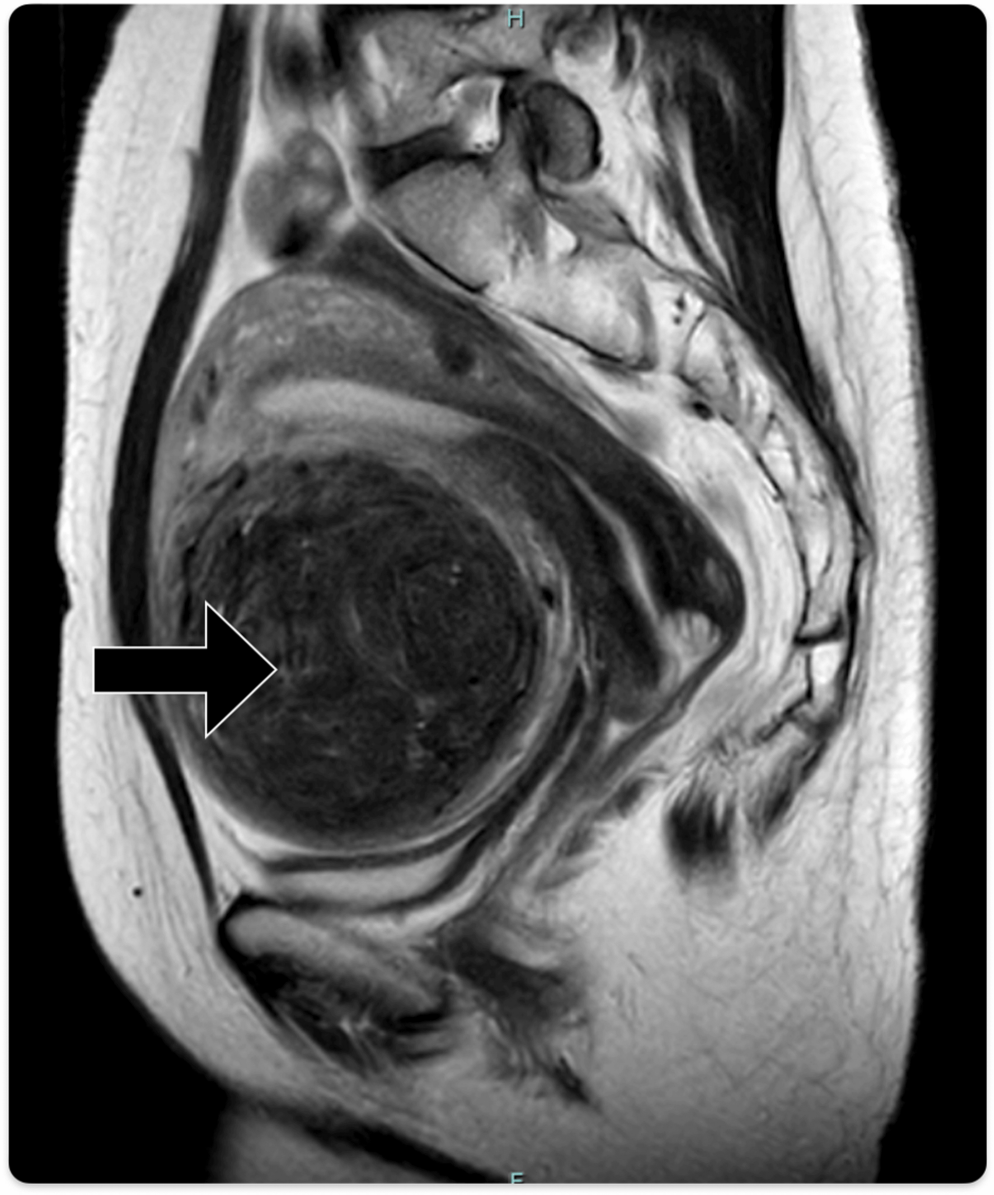
Pre-TLH T2-weighted MRI (sagittal view) demonstrating a low-intensity 12 cm mass (arrow) located on the anterior wall of the uterus. TLH: total laparoscopic hysterectomy

The patient was a 51-year-old sexually active woman with no pregnancies. Six years prior, she had undergone TLH for uterine leiomyoma. There was no notable family history or regular medication use. Pre-TLH MRI had revealed a 12 cm leiomyoma-like tumor on the anterior uterine wall without any signs suggestive of sarcoma (Figure 1).

**Figure 2:**
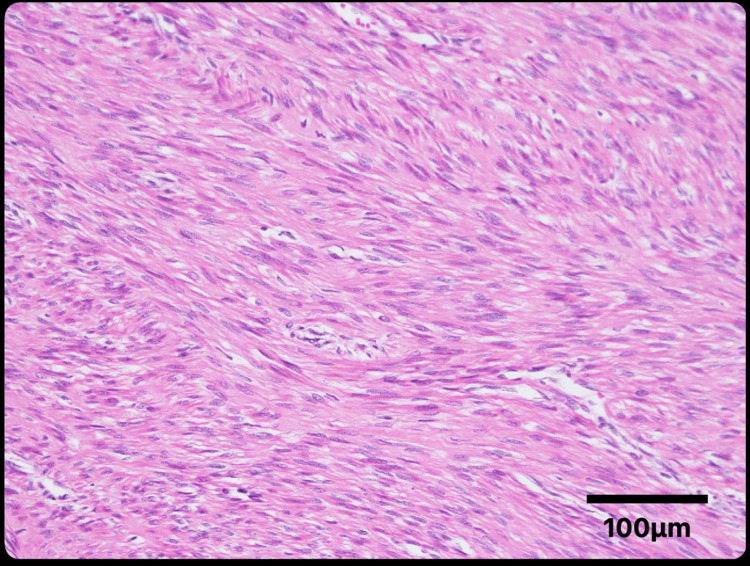
Histopathological image after TLH showing densely packed spindle cells with eosinophilic cytoplasm and no atypia, consistent with a diagnosis of leiomyoma TLH: total laparoscopic hysterectomy

TLH and bilateral salpingectomy were performed. The specimen was morcellated using a scalpel within the abdominal cavity and subsequently retrieved through a vaginal approach without the use of a retrieval bag. The specimen weighed 1147 grams and histopathological examination showed densely packed spindle cells with eosinophilic cytoplasm and no atypia, consistent with a diagnosis of leiomyoma (Figure 2). There were no malignant findings, and no abnormalities were detected in the cervix, endometrium, or fallopian tubes. 

**Figure 3:**
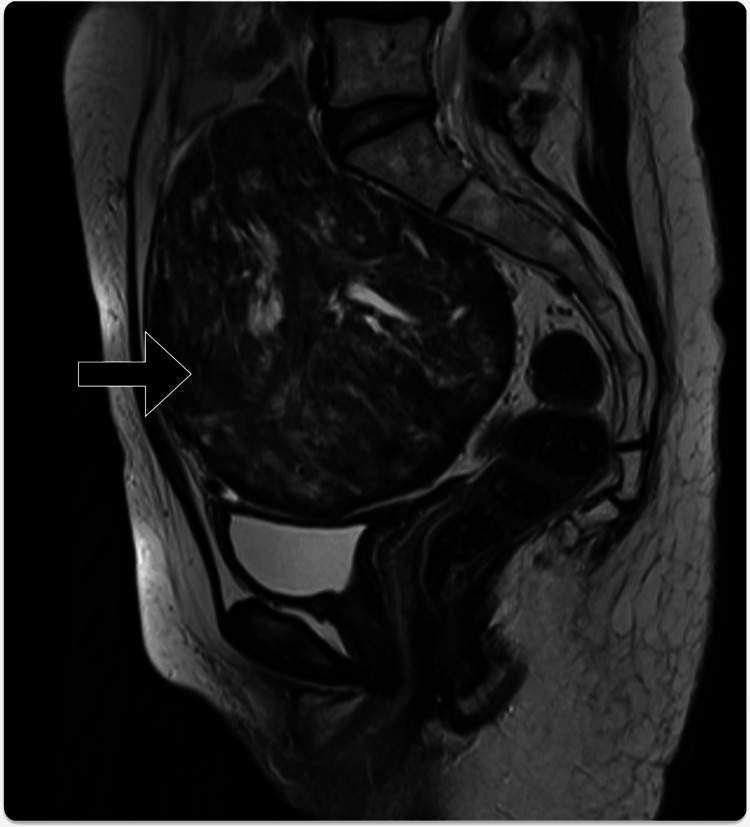
T2-weighted MRI showing a 21×12×15 cm tumor in the pelvic cavity

Six years after the surgery, the patient presented with a complaint of a lower abdominal mass. There were no additional symptoms, such as abdominal pain or gastrointestinal complaints. MRI conducted showed a 21×12×15 cm tumor in the pelvic cavity, with isointense signals to the muscle on T1-weighted images and a heterogeneous appearance of low to moderate signal intensity with crack-like high signals on T2-weighted images. No abnormal diffusion high signals were observed on the diffusion-weighted imaging, and both ovaries were normal (Figure 3). Considering the possibility of a leiomyoma originating from the peritoneum or mesentery, a differentiated sarcoma, or a GIST, the patient was referred to our hospital for further examination and treatment.

**Figure 4:**
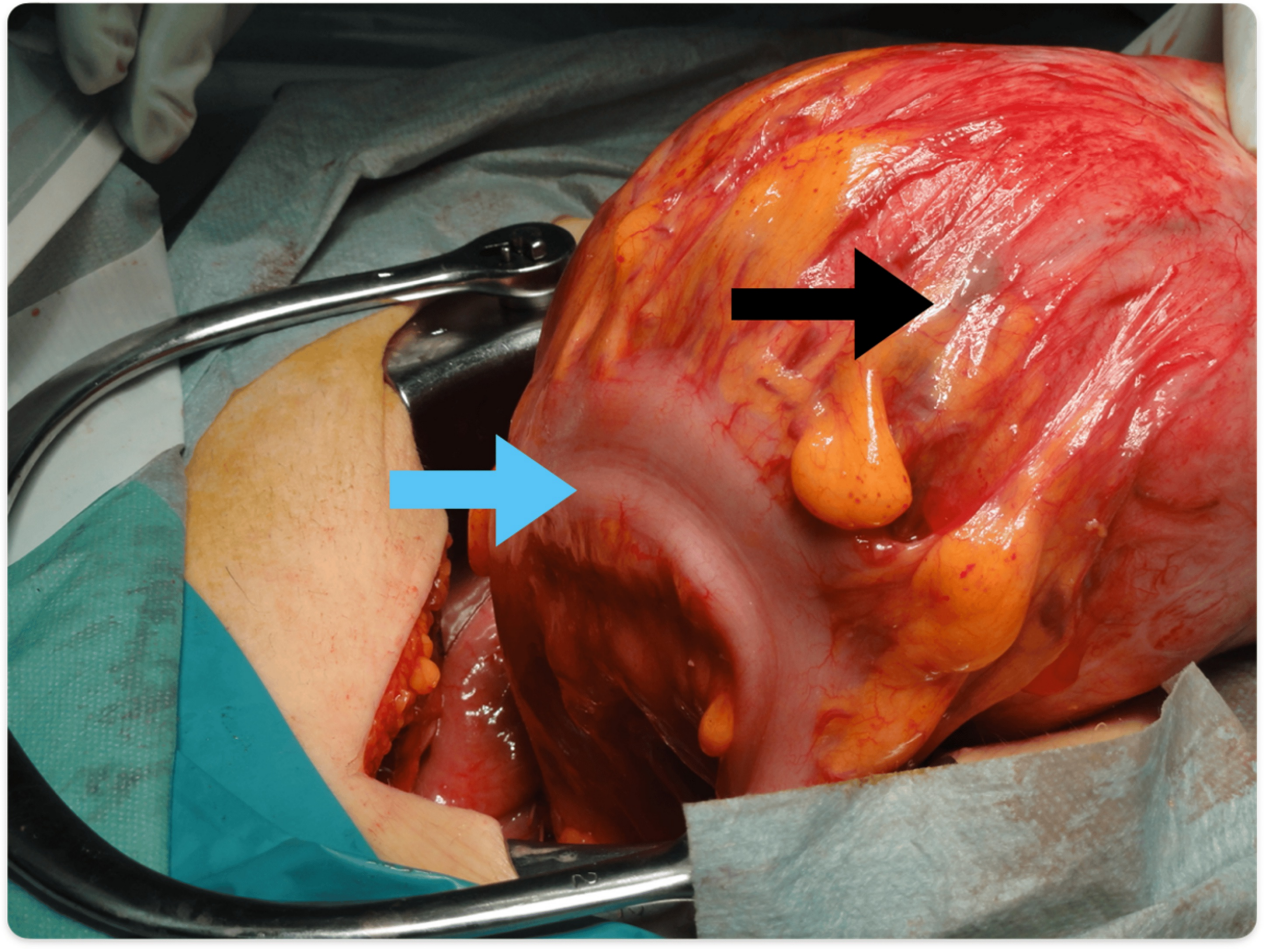
Intraoperative findings of PL The PL (black arrow) originated from the sigmoid mesentery and was firmly adhered to the sigmoid colon (blue arrow). PL: parasitic leiomyoma

Preoperative investigations did not show elevation of tumor markers (lactate dehydrogenase (LDH) 140 U/L, carcinoembryonic antigen (CEA) 0.7 ng/mL, cancer antigen (CA) 19-9 5.1 U/mL, CA125 5.7 U/mL, alpha-fetoprotein (AFP) 1.1 ng/mL, squamous cell carcinoma (SCC) 1.4 ng/mL, neuron-specific enolase (NSE) 8.7 ng/mL) or abnormal findings in vaginal stump cytology. The tumor was diagnosed as of unknown primary origin, and an open tumor excision was performed. Due to the risk of ureteral injury in retroperitoneal tumors, double J ureteral catheters were placed in both ureters during surgery. The tumor was firmly adhered to the mesentery of the sigmoid colon (Figure 4). Under the support of a gastrointestinal surgeon, adhesions were dissected using an energy device, and the tumor was removed. The ascitic fluid that had accumulated in the abdominal cavity was submitted for cytological examination. We did not perform a frozen section cytology, as it is challenging to reliably distinguish between benign and malignant findings in this context. No other tumors were detected within the abdominal cavity. The postoperative course was uneventful, and the patient was discharged on postoperative day 7.

**Figure 5:**
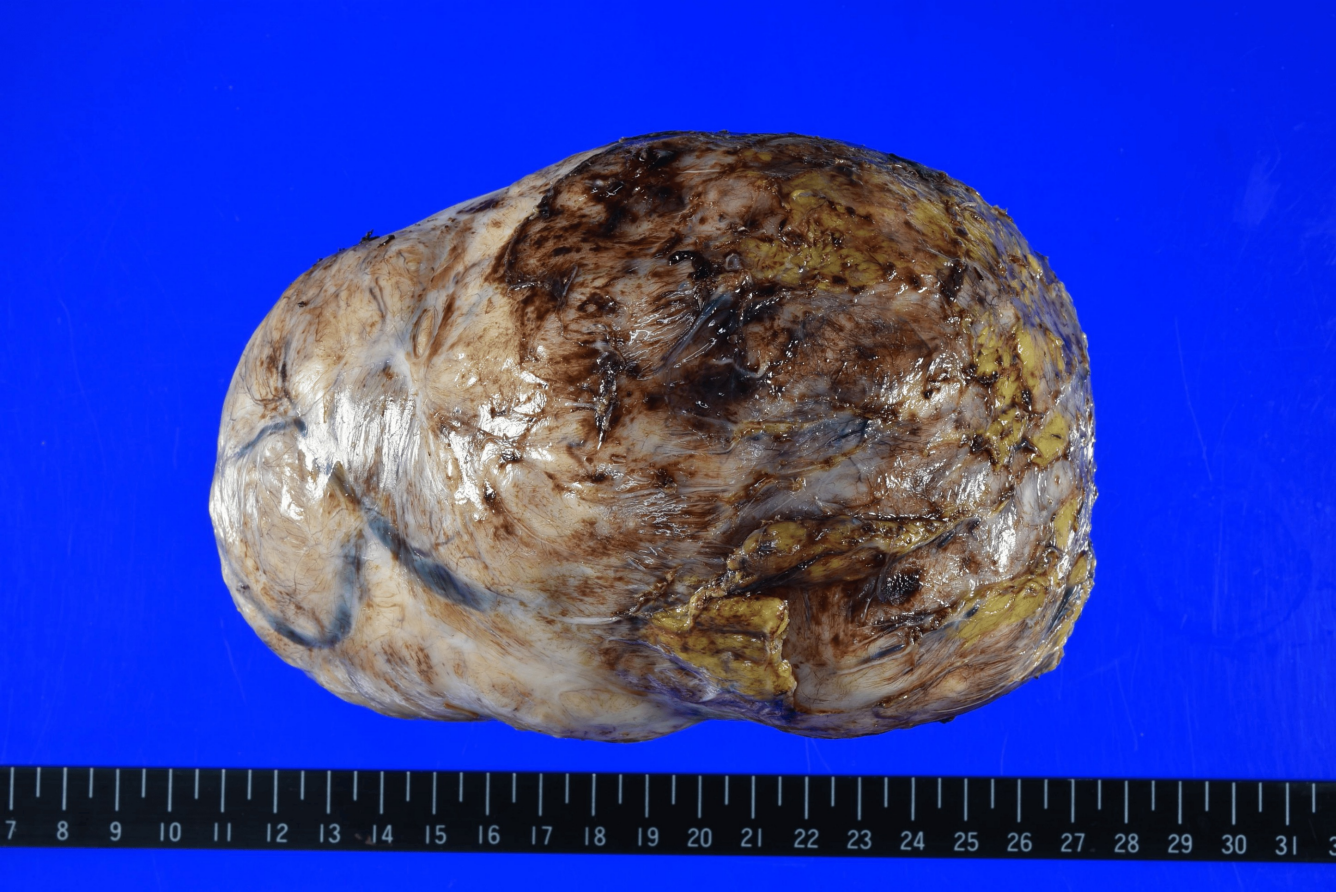
Excised PL specimen weighing 1020 g and measuring 16×12×10 cm PL: parasitic leiomyoma

**Figure 6:**
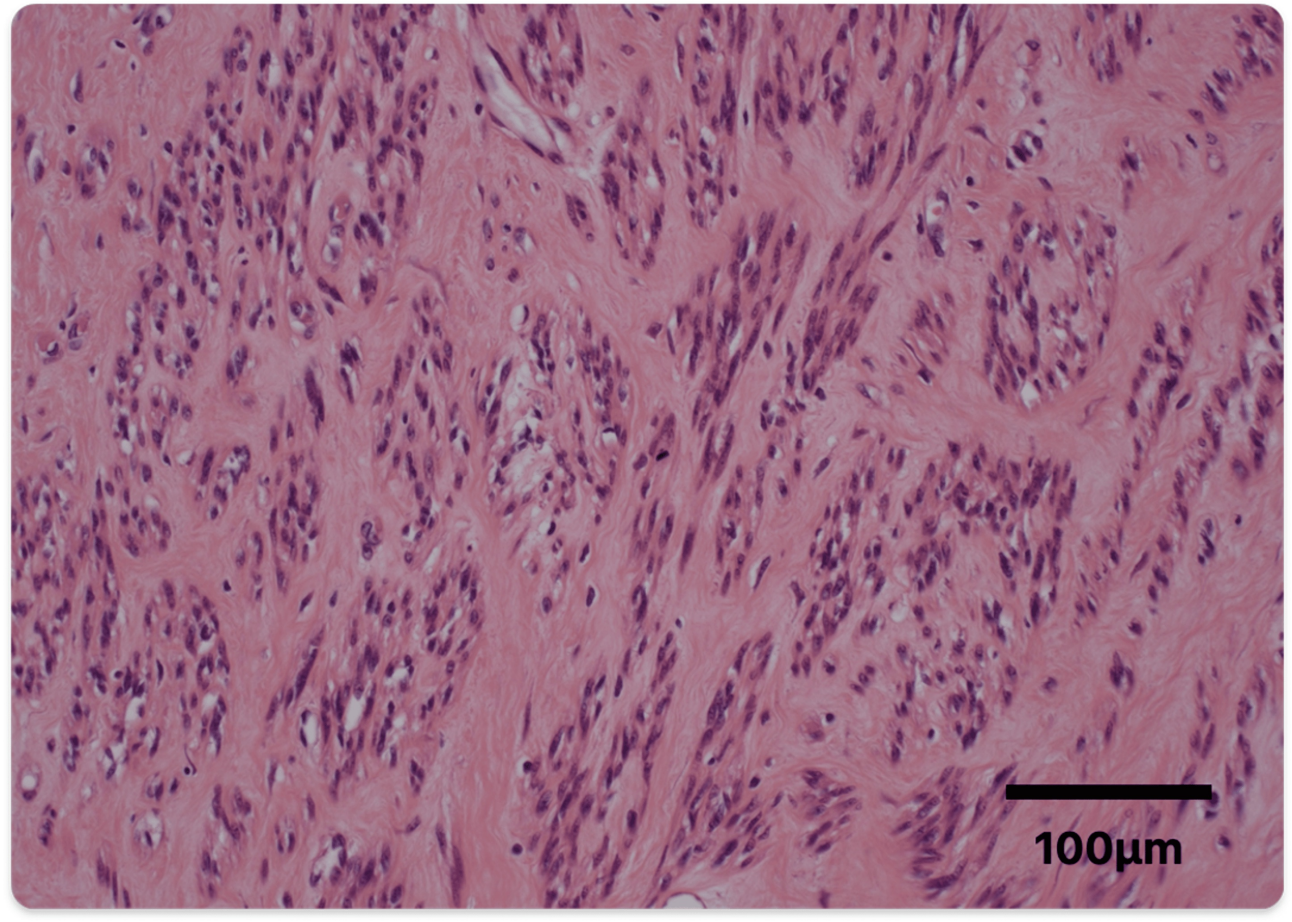
Histopathological image of PL The tissue was characterized by eosinophilic spindle cells arranged in fascicles and whorls. The nuclei were elongated and oval with very few mitotic figures observed. PL: parasitic leiomyoma

The surgical specimen weighed 1020 g and measured 16×12×10 cm (Figure 5). The results of the ascites cytology were available three days later and showed no evidence of malignancy. Histopathologically, the tissue was characterized by eosinophilic spindle cells arranged in fascicles and whorls. The nuclei were elongated and oval with very few mitotic figures observed (Figure 6). The diagnosis was consistent with leiomyoma, and there were no histological differences between the initial uterine myoma and the parasitic one, both shared identical pathological characteristics. The postoperative course was favorable with no recurrence.

## Discussion

PLs are rare uterine leiomyomas that develop outside the uterus, often in the abdominal cavity [11]. Their incidence has increased in recent years, possibly due to laparoscopic morcellation during myomectomy or hysterectomy [12].

Three mechanisms have been proposed for the development of PL. The first involves pedunculated subserosal leiomyomas that adhere to surrounding tissues and acquire a blood supply, after which the stalk disappears [13]. The second mechanism is disseminated peritoneal leiomyomatosis (DPL), where leiomyomas develop due to pseudotransformation of the peritoneum [14]. The third occurs when leiomyomas are morcellated during retrieval, and the fragments become dispersed and implant within the abdominal cavity [13].

In the current case, the leiomyoma grew over six years following TLH, ruling out the possibility of development from the adhesion of a subserosal leiomyoma. Additionally, DPL typically presents as multiple occurrences across the peritoneum, making it unlikely in the instance of a solitary, large leiomyoma as seen in the present case. Therefore, it is likely that this case involved fragments morcellated during TLH which were implanted and developed in the mesentery.

A search on PubMed for "parasitic myoma and leiomyoma mesentery" yields nine reports [2,15-22], of which six cases involved PL development following surgery for uterine leiomyomas [17-22]. However, there were no reported cases of PL developing after TLH.

In the present case, a large PL developed in the mesentery of the sigmoid colon following TLH. Its origin was challenging to determine, complicating the diagnosis as it closely resembled malignant retroperitoneal tumors such as leiomyosarcoma, GIST, and others. If the tumor had been malignant, a combined resection of the sigmoid colon, rather than a simple tumor excision, might have been necessary. Based on the patient's surgical history and preoperative examination results, PL was strongly suspected, leading to the decision to perform a simple tumor excision. This approach ultimately resulted in the best possible outcome.

The retroperitoneum can host a wide spectrum of pathologies, including a variety of rare benign tumors and malignant neoplasms that can be either primary or metastatic lesions [23]. To prevent the occurrence of PL, IBM should be standard practice. Further accumulation of cases is needed to enhance our understanding and management of PL.

## Conclusions

We reported a case of large PL in the mesentery following TLH for uterine leiomyoma. In this case, it has been deduced that morcellation within the peritoneal cavity caused fragments of the uterine leiomyoma to implant and grow in the mesentery. When encountering a solid tumor of unknown cause after a gynecologic surgical procedure, a parasitic myoma must be included in the differential diagnosis. Further accumulation of cases is needed to enhance our understanding and management of PL.
